# Ready meals, especially those that are animal-based and cooked in an oven, have lower nutritional quality and higher greenhouse gas emissions and are more expensive than equivalent home-cooked meals

**DOI:** 10.1017/S1368980023000034

**Published:** 2023-03

**Authors:** Magaly Aceves-Martins, Philippa Denton, Baukje de Roos

**Affiliations:** The Rowett Institute, University of Aberdeen, Aberdeen AB25 2ZD, UK

**Keywords:** Ready meals, Home-cooked meals, Nutritional quality, Cost, GHGE

## Abstract

**Objective:**

To examine whether ready meals and equivalent home-cooked meals differ in nutritional quality indicators, greenhouse gas emissions (GHGE) and cost.

**Design:**

We performed a cross-sectional analysis of meal data from the National Diet and Nutrition Survey (NDNS) nutrient databank (2018/19). Additional data on nutrient composition, cost and cooking-related GHGE were calculated and compared between fifty-four ready meals and equivalent home-cooked meals.

**Setting:**

The UK.

**Participants:**

Not applicable.

**Results:**

Ready meals, overall and those that were animal-based, had significantly higher levels of free sugar compared with equivalent home-cooked meals (*P* < 0·0001 and *P* < 0·0004, respectively). Animal-based ready meals had significantly higher levels of GHGE (*P* < 0·001), whereas the cost of ready meals, overall, was significantly higher (*P* < 0·001), compared with equivalent home-cooked meals. Animal-based meals, whether ready meals or equivalent homemade meals, had significantly higher levels of protein (*P* < 0·0001), contained significantly more kilocalories (*P* = 0·001), had significantly higher levels of GHGE (*P* < 0·0001) and were significantly more expensive (*P* < 0·0001), compared with plant-based meals. Overall, plant-based meals home-cooked on the gas or electric stove had the lowest GHGE and cost, whereas animal-based oven-cooked ready meals had the highest levels of GHGE and were most expensive.

**Conclusions:**

Ready meals have lower nutritional quality and higher GHGE and are more expensive than equivalent home-cooked meals, especially those meals that are animal-based and prepared in an oven.

## Graphical abstract

**Figure:**
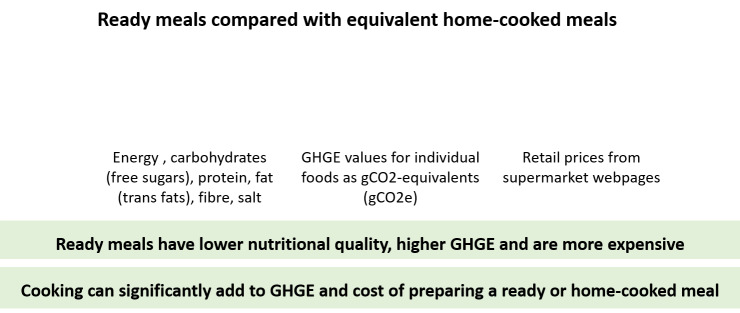

Ultra-processed foods and formulations of ingredients, primarily of exclusive industrial use and typically created by a series of industrial techniques and processes, are increasingly dominating our food supply chains. Ultra-processed foods are mostly ready-to-consume, hyper-palatable and profitable branded products designed to displace other food groups^(1)^. Consumption of ultra-processed foods has been associated with a range of detrimental health outcomes in epidemiological studies, including an increased risk of all-cause mortality, CVD, hypertension, metabolic syndrome, overweight and obesity^(2)^. Whilst there is increasing evidence that consumption of ultra-processed foods may be damaging to human health, its environmental impacts are poorly quantified. Current evidence only considers the effects of primary commodities used for their production rather than capturing the overall impact of ultra-processed foods from farm to fork, including processing, packaging and distribution^(3)^.

Many ready meals, defined as pre-prepared main courses sold in a pre-cooked form that only requires pre-heating prior to consumption, can be classified as ultra-processed foods. The UK has one of the largest ready meal markets globally, with a market value of over £3·9 billion^(4)^. It is estimated that 88 % of the UK adult population eat ready meals, with two out of five people eating them every week^(5)^. Chilled ready meals make up 70 % of the UK ready meals market share, with frozen meals occupying the remaining 30 %^(6)^. Some of the main drivers for the steady rise in the purchase and consumption of ready meals include time scarcity in modern life, more women in the workplace, varying eating times, lack of cooking skills or dislike of cooking, and a growing number of single households^(7,8)^.

Like most ultra-processed foods, ready meals are generally energy-dense and contain higher levels of low-cost ingredients such as saturated and *trans*-fats, refined starches, free sugars and salts, whilst being low in fibre and micronutrients^(1)^. Besides their poor nutritional profile, another main concern is that greenhouse gas emissions (GHGE) from the consumption of ready meals in the UK currently contribute 15·7 % of the total annual GHGE from the UK food and drink sector^(6)^. Also, it is estimated that ready meals represent 8 % of the total per capita carbon budget related to food production for the climate target of limiting warming to 2°C^(6)^. A few studies have compared the environmental effects of consuming ready meals *v*. home-cooked meals, with one study finding that the environmental impact of homemade meals was lower because of avoidance of meal manufacturing, reduced refrigeration and a lower amount of waste in the life cycle of the homemade meal^(9)^. However, another study noted that the differences in environmental impact between both ready and homemade meal options were small and highlighted that homemade meals had a higher environmental impact than semi-prepared or ready-to-eat meals^(10)^.

This study aimed to assess how ready meals compared with equivalent home-cooked meals in terms of nutritional quality indicators and GHGE, but also in terms of cost, in main meals consumed in the UK. Indeed, affordability is an essential determinant of food choice by consumers in the UK and a pivotal contributor to socio-economic inequalities when considering the healthiness of food and drink choices^(11–14)^.

## Methods

### Data

We performed a secondary data analysis using the National Diet and Nutrition Survey (NDNS) nutrient database year 11 (2018/19)^(15–17)^. The NDNS nutrient databank contains compositional data from the nearly 6000 foods, drinks and prepared dishes available in the UK, including home-cooked and ready meals. Of these, we selected all main course meals, chilled or frozen, that needed to be heated prior to consumption, sold within a container, and had an equivalent home-cooked version in the NDNS nutrient database. As a result, we included fifty-four main courses with data on nutrient profile, and on frequency of consumption over 4 d, in 444 participants (Table 1).

**Table 1:** Ready meal dishes, and home-cooked equivalent dishes, including frequency of consumption, GHGE and cost per dish

Ready meals				Home-cooked equivalent meals			
Food name	Frequency*	GHGE†	Cost‡	Food name	Frequency*	GHGE†	Cost‡
Animal-based							
Beef and potato pie	2	1543	0·51	Beef and potato pie 2 crusts	0	433	0·96
Beef stew and dumplings frozen or chilled ready meal	1	1400	0·87	Beef stew and dumplings	0	390	0·56
Beef stir fry with green peppers and black bean sauce	0	1280	1·36	Beef stir fry	0	1150	0·55
Minced beef pie purchased	4	1200	0·62	Minced beef pie top pastry	0	706	0·96
Steak pie, short crust, purchased	3	1200	0·84	Steak pie pastry top only	0	797	0·19
Cornish pasty purchased	10	1200	0·44	Cornish pasty homemade	0	508	0·14
Corned beef pasty purchased	0	1200	0·66	Corned beef pasty	0	508	0·14
Lamb bhuna purchased	0	1180	1·00	Lamb curry (no potatoes) with onions and curry pas	0	1019	1·20
Tagliatelle carbonara ready meal	1	1110	0·37	Spaghetti carbonara	0	431	0·74
Chilli con carne no rice ready meal	0	1070	0·37	Chilli con carne minced beef kid beans and tin tom	2	433	0·16
Cottage pie, frozen/chilled beef	4	1040	0·64	Cottage pie	0	285	0·22
Cottage pie, reduced fat, ready meal	0	1040	0·28	Cottage pie with lean minced beef, potatoes and butter	0	285	0·22
Lasagne beef, ready meal	5	1000	0·66	Lasagne homemade	1	497	0·24
Lasagne, reduced fat, ready meal	1	1000	0·66	Lasagne made with extra lean mince	0	497	0·24
Beef curry frozen/chilled ready meal no rice	1	900	0·25	Beef curry with cream or coconut sauce	0	242	0·32
Shepherd’s pie, lamb, ready meal	0	880	0·65	Shepherd’s pie homemade with minced lamb	0	485	0·48
Beef hot pot with pots ready meal	1	810	0·56	Beef hot pot made with stewing steak carrots cab	0	498	0·56
Moussaka ready meal chill/frozen/long life	1	670	0·87	Moussaka with aubergines homemade	0	678	0·36
Chicken curry frozen chilled no rice	3	670	0·2	Chicken curry homemade	6	553	0·62
Lamb hot pot with potatoes ready meal	0	670	0·87	Lamb hot pot	0	590	0·48
Lemon chicken	0	530	1·00	Lemon chicken – chicken breasts in sauce	1	463	1·25
Chicken chow mein ready meal	3	530	0·70	Chicken Chow Mein	3	401	0·87
Quiche, meat-based, Quiche Lorraine not low fat	12	491	0·58	Quiche Lorraine not wholemeal	0	611	0·52
Fishcakes, salmon, retail, coated in breadcrumbs, baked/grilled	5	460	0·74	Salmon fishcakes grilled	0	209	1·20
Smoked haddock chowder, for example M&S	0	460	0·44	Fish and seafood chowder	2	256	0·58
Tuna and pasta bake ready meal	0	460	0·68	Tuna and pasta bake	0	498	0·39
Sweet and sour pork frozen ready meal no rice	0	460	1·10	Sweet and sour pork	0	360	0·58
Chicken and sweetcorn soup	1	410	0·21	Chicken and veg soup with carrot potato and onion	0	147	0·19
Chicken pie frozen/chilled individual two crusts	7	400	0·37	Chicken pie 2 crusts	0	525	0·62
Chicken in white sauce ham mushroom and rice	0	400	0·85	Chicken and mushrooms in white wine sauce	1	507	0·53
Chicken and pasta bake with broccoli, low fat	0	400	0·68	Chicken and broccoli pasta bake	0	650	0·65
Chicken casserole chicken in tomato/gravy/sauce and vegetables	0	400	0·62	Chicken and vegetable casserole with olive oil	0	345	0·23
Fisherman’s pie (white fish) retail	2	400	0·5	Fisherman’s pie (potato based) with cod and prawns	0	265	0·96
Fisherman’s pie reduced calorie and fat retail	0	400	0·5	Fisherman’s pie with prawns and smoked haddock	0	265	0·96
Tuna and red pepper fish cakes	0	329	1·0	Tuna and potato fish cakes	0	215	0·21
Plant-based							
Macaroni cheese ready meal low fat	4	1110	0·20	Macaroni cheese semi skim milk and reduced fat spread	1	407	0·25
Macaroni cheese purchased	3	1110	0·22	Macaroni cheese with butter and semi-skimmed milk	0	407	0·25
Broccoli and stilton soup, premium, chilled carton	0	1110	0·11	Broccoli and cheese soup homemade	0	203	0·18
Quiche, cheese and onion, purchased	13	491	0·58	Cheese and onion quiche homemade	0	479	0·38
Cheese and vegetable quiche purchased	1	491	0·57	Cheese and tomato quiche	1	476	0·38
Quiche, vegetable only, no cheese, purchased	1	491	0·58	Cauliflower and broccoli quiche	0	658	0·38
Mushroom soup, premium, chilled, carton	1	480	0·18	Homemade mushroom soup	0	107	0·18
Vegetable curry, ready meal, no rice	0	280	0·66	Vegetable curry	3	123	0·29
Ross veg chow mein stir-fried in olive oil	0	270	0·54	Vegetable Chow Mein	0	316	0·25
Vegetable lasagne purchased	0	260	0·87	Vegetable lasagne homemade	1	241	0·32
Vegetable bake purchased ready meal	0	260	0·37	Vegetable bake with carrots, broccoli, potatoes and cheese sauce	0	120	0·21
Cauliflower cheese: ready meal purchased standard	2	220	0·67	Cauliflower cheese (whole milk)	0	279	0·20
Cauliflower cheese: healthy range ready meal purchased	0	220	0·67	Cauliflower cheese with butter and semi-skimmed milk	0	279	0·20
Vegetable shepherd’s pie – purchased ready meal	0	220	0·27	Vegetable shepherd’s pie	0	265	0·21
Spinach and potato curry purchased or takeaway	1	180	0·66	Spinach and potato curry with tomatoes and onion	0	123	0·29
Vegetable soup carton	6	150	0·18	Soup vegetable	0	56	0·21
Carrot and coriander soup, purchased	2	150	0·12	Carrot and onion soup homemade	0	58	0·04
Cream of tomato soup, carton	1	150	0·25	Tomato soup with cream, homemade	0	110	0·19
Ratatouille frozen purchased	0	120	0·66	Ratatouille homemade	1	155	0·25

* Frequency of consumption across all participant’s (*n* 444, NDNS 2018/2019) 4-d dietary recalls.

† GHGE per 100 g of product up to supermarket shelf.

‡ Cost per 100 g of product.

### Nutritional quality indicators

Relevant indicators of nutritional quality, including total kilocalories, carbohydrates (including free sugars), protein, fat (including *trans*-fats), fibre and salt, were selected based on previous publications reporting on differences in nutritional quality between ready-made and home-cooked meals^(18,19)^. These indicators were estimated per 100 g of a meal.

### Greenhouse gas emissions

GHGE values for individual foods and ready meals expressed as gCO2 equivalents (gCO2e) were obtained from a range of open-access sources, including academic studies, retailers and producers published between 2008 and 2016^(20,21)^, added to the NDNS nutrient databank^(21,22)^. GHGE values were based on the emissions of six greenhouse gases which were converted into an equivalent amount of carbon dioxide (CO2 equivalent or CO2e), based on the relative global warming impact of each gas, and the final carbon footprint was expressed as the weight of carbon dioxide^(20)^. The climate metric used to aggregate the GHGE measurements into CO2e were those reported by Department for Environment Food and Rural Affairs, UK^(23)^. GHGE values from studies using complete cradle-to-grave life cycle analysis (LCA)^(20)^, obtained following the international PAS 2050 standard^(24)^, were selected where possible. We identified CO2e for 153 food and drink items in the NDNS nutrient databank, and where a GHGE value for a specific item was not available, reasonable substitute data were discussed and imputed by a team of three nutrition scientists, based on the food type, food group and compositional similarity of the products.

To estimate the GHGE for home-cooked meals, we estimated GHGE of the raw ingredients, establishing the weight of each ingredient and the weight of the whole cooked meal using Nutritics, which is nutrition management software for recipe and menu management, food labels, diet and activity analysis, and meal planning (Nutritics Ltd). Based on BBC Good Food^(25)^ and Sainsbury’s recipes^(26)^, we established cooking methods and times. For home-cooked meals requiring more than one cooking method, GHGE data for each cooking method were added together. In addition, we recorded the longest cooking time suggested for the frozen versions of ready meals. If there was more than one suggested cooking method (e.g. oven and microwave), data for both methods were recorded separately.

To estimate the full GHGE until serving the meal, we combined the GHGE from the recipes’ ingredients or ready meals (value up to the supermarket shelf), which include emissions due to land use change, farm-related emissions, animal feed, processing, transport, retail and packaging) with GHGE produced by the different cooking methods. For the latter, GHGE of cooking appliances were based on manufacturer information^(27)^ and adjusted to the conversion factors provided by the UK government in 2021^(28)^ and cooking time (Equation 1):
(1)

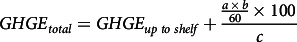




where *a* is the cooking time, *b* is the GHGE of cooking appliances based on manufacturer information and adjusted to the conversion factors given by the UK government 2021, and *c* is the weight of the recipe or ready meal product.

### Cost

For ready meals, we used the retail prices from the supermarket/products webpages (last accessed in January 2022) to estimate the total cost per 100 g. We used the price per serving of home-cooked meals published on either the BBC Good Food^(25)^ or Sainsbury’s^(26)^ recipes website (last accessed on November 2021). If prices were not available, we estimated the cost of the raw ingredients established the weight of each ingredient and the weight of the whole cooked meal using Nutritics (Nutritics Ltd). We added the costs from each ingredient to get the total cost for the meals and then estimated the total cost per 100 g. Our analysis did not include the costs for reheating or cooking the meals.

### Analysis

We analysed nutritional quality and estimated total GHGE (values up to supermarket shelf plus GHGE after cooking) and cost for each of the fifty-four ready meals and fifty-four equivalent home-cooked meals. Distributions of data were analysed visually, and Shapiro–Wilk tests were performed to test normality for each outcome (online Supplementary Table 2). These tests suggested significant non-normality; hence, non-parametric tests (median differences) were selected for analysis. Mann–Whitney tests were used to compare nutritional values, GHGE and cost between ready meals and their equivalent home-cooked meals. The percentage change was estimated from GHGE values up to supermarket shelves and after cooking, and statistical significance was assessed through paired *t* test analysis. We expressed data in medians and interquartile ranges (IQR). Statistical significance was estimated at *P* < 0·05, but a Bonferroni correction was included in the analysis to control the family-wise error. We performed a sub-analysis of plant- *v*. animal-derived meals because of the published evidence on differences in nutritional quality and GHGE between these meals^(29)^.

Data were visualised with Tableau software, and statistical analysis was performed in R software using the libraries ‘*ggthemes*’, ‘*tidiverse*’ ‘for data visualisation and graphs), ‘*dplyr*’ (for testing normality), ‘*psych*’ and ‘*pastecs*’ (for descriptive statistics).

## Results

Of the fifty-four ready meal and home-cooked meal main courses we identified in the NDNS nutrient database (Table 1), 65 % were animal-based and 35 % were plant-based. Ready meals, overall and those that were animal-based had significantly higher levels of free sugar per 100 g of product, compared with equivalent home-cooked meals (*P* < 0·0001 and *P* < 0·0004, respectively). Animal-based ready meals had significantly higher levels of GHGE (up to supermarket shelf) per 100 g of product (*P* < 0·001), whereas the cost of ready meals, overall, was significantly higher per 100 g of product (*P* < 0·001), compared with equivalent home-cooked meals (Table 2). Across ready meals and equivalent home-cooked meals, animal-based meals had significantly higher levels of protein (*P* < 0·0001), contained significantly more kilocalories per 100 g of product (*P* = 0·001), had significantly higher levels of GHGE (up to supermarket shelf) per 100 g of product (*P* < 0·0001), and were significantly more expensive (*P* < 0·0001), compared with plant-based meals (Table 2).

**Table 2:** Differences in nutritional quality, greenhouse gas emissions between ready meals and equivalent home-cooked meals, and between animal-based meals and plant-based meal variants

		Meal origin	Ready meals		Home-cooked meals		P_rm-hc_	P_ab-pb_
			Interquartile range	IQR	Interquartile range	IQR		
Nutritional quality indicators	Total carbohydrates (g/100 g)	All meals (*n* 54)	11·3	10·3	10·3	10·7	0·27	–
		Animal-based meals (*n* 35)	11·7	9·25	10·8	10·9	0·33	0·24
		Plant-based meals(*n* 19)	9·6	10·7	7·3	9·25	0·64	
	Free sugars (g/100 g)	All meals (*n* 54)	0·5	1·1	0·1	0·4	0·0001*	–
		Animal-based meals (*n* 35)	0·5	0·9	0	0·3	0·0004*	0·87
		Plant-based meals(*n* 19)	0·8	1·1	0	0·5	0·08	
	Total protein (g/100g)	All meals (*n* 54)	6·7	4·7	8·9	5·3	0·01	–
		Animal-based meals (*n* 35)	8·1	3·7	10·5	2·7	0·002	< 0·0001*
		Plant-based meals(*n* 19)	3·5	3·3	4·1	6·05	0·39	
	Total fat (g/100g)	All meals (*n* 54)	4·7	7·5	6·4	5·6	0·10	–
		Animal-based meals (*n* 35)	4·8	7·1	6·4	5·5	0·15	0·22
		Plant-based meals(*n* 19)	3·6	5·3	6·3	3·2	0·52	
	*Trans*-fat (g/100 g)	All meals (*n* 54)	0·1	0·1	0·1	0·2	0·30	–
		Animal-based meals (*n* 35)	0·1	0·1	0·1	0·2	0·16	0·42
		Plant-based meals(*n* 19)	0·12	0·9	0·1	0·2	0·92	
	Fibre (g/100 g)	All meals (*n* 54)	1·5	0·7	1·2	0·6	0·06	–
		Animal-based meals (*n* 35)	1·4	0·7	1·1	0·7	0·09	0·09
		Plant-based meals(*n* 19)	1·6	0·5	1·3	0·5	0·38	
	Salt (mg/100 g)	All meals (*n* 54)	600	303·7	445	482·5	0·006	–
		Animal-based meals (*n* 35)	665	276·2	482·5	559	0·02	0·02
		Plant-based meals(*n* 19)	500	213·7	415	216·2	0·17	
	Energy (kcal/100 g)†	All meals (*n* 54)	116·5	78·8	128·5	89·2	0·29	–
		Animal-based meals (*n* 35)	124	85	140	79·7	0·21	0·001*
		Plant-based meals(*n* 19)	86	78·5	96·5	65	0·63	
GHGE	Total GHGE up to shelf (gCO2e/100 g)	All meals (*n* 54)	491	640	404	259·2	0·002	–
		Animal-based meals (*n* 35)	670	630	487	197	0·001*	< 0·0001*
		Plant-based meals(*n* 19)	260	291	263·2	169·6	0·15	
Cost	Total cost (GBP/100 g)	All meals (*n* 54)	0·62	0·31	0·32	0·36	0·001*	–
		Animal-based meals (*n* 35)	0·62	0·35	0·54	0·47	0·13	< 0·0001*
		Plant-based meals(*n* 19)	0·58	0·43	0·23	0·09	0·006	

GHGE, greenhouse gas emissions up to supermarket shelf; gCO2e, gCO2 equivalents; GBP, Great British pound £; P_rm-hc_, *P*-value of difference between ready meals and equivalent home-cooked meals; P_ab-pb_, *P*-value of difference between animal-based and plant-based meals, across ready and home-cooked meals.

Data represent medians and interquartile range (IQR).

* Statistical significance, adjusted using Bonferroni correction, estimated at a *P*-value < 0·0016.

**Fig. 1:**
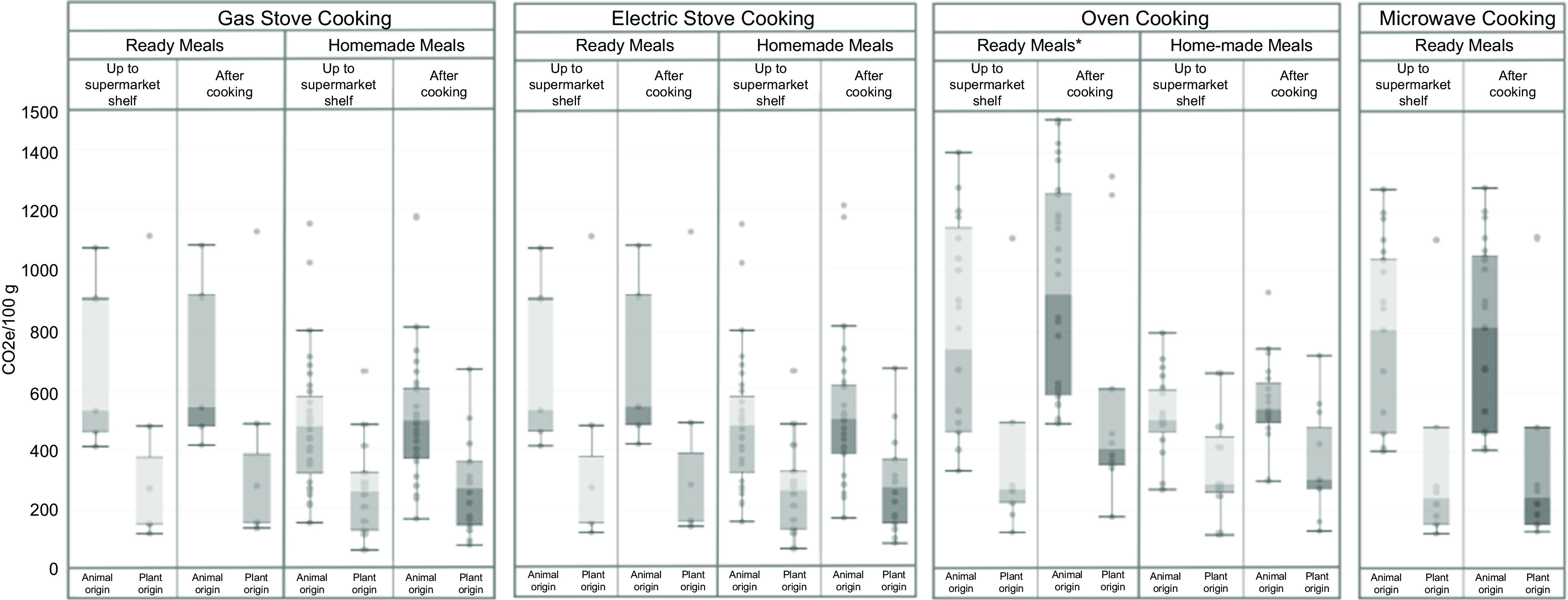
Distribution of GHGE per cooking method, type and origin of the meals. gCO2e,gCO2 equivalents. Boxplots presenting median values. Whisker’s extension to data within 1·5 times the interquartile ranges. Darker grey within and bar shows the lower whisker, and the lighter grey, the higher whisker. No homemade meal recipe required the use of microwave, and hence no comparison among ready meals and homemade meals was feasible for this cooking method. Oven-cooked ready meals had significantly higher levels of GHGE compared with equivalent home-cooked meals (*P* < 0·05). Cooking generally resulted in a significant increase in GHGE across all meals and cooking methods (*P* < 0·05). Across meals and cooking methods, GHGE values after cooking meals that were animal-based were significantly higher than GHGE values for plant-based meals (*P* < 0·005) (Table 3). Gas and electric stove-cooked meals: *n* 62 (twelve ready meals of which five animal-based and seven plant-based; and fifty home-cooked meals of which thirty-three animal-based and seventeen plant-based). Microwave-cooked meals: thirty-nine ready meals of which twenty-five animal-based and fourteen plant-based. Oven-cooked meals: *n* 77 (forty-six ready meals of which fifty-two animal-based and twenty-five plant-based; and thirty-one home-cooked meals of which twenty animal-based and eleven plant-based)

Stove and microwave cooking of ready meals and equivalent home-cooked meals generally resulted in a small increase in GHGE, adding on average 1–4 % to ‘up to supermarket shelf’ GHGE. Oven cooking of ready meals and equivalent home-cooked meals resulted in much higher increases in GHGE, adding on average 19 and 8 %, respectively, to ‘up to supermarket shelf’ GHGE. Ready meals, overall and those that were animal-based, had significantly higher levels of GHGE, after cooking, compared with equivalent home-cooked meals (*P* < 0·0005). Levels of GHGE, after cooking, were significantly higher for animal-based meals than for plant-based meals, (*P* < 0·0027), across meals and cooking methods (Fig. 1, Table 3).

**Table 3:** Differences in greenhouse gas emissions between ready meals and equivalent home-cooked meals, and between animal-based meals and plant-based meal variants, by cooking method

Cooking method	Origin of meals	GHGE value	Ready meals		Homemade meals		P_rm-hc_	P_ab-pb_
			Median	IQR	Median	IQR		
Gas stove cooked (Total gCO2e/100 g)	All meals (*n* 62) Ready meals (*n* 12) Home-cooked meals (*n* 50)	Up to supermarket shelf	435	472·5	395·5	252·2	0·72	–
		After cooking	448·0	474·4	404·9	237·3	0·85	
		GHGE % increase due to cooking	2·9 %		2·3 %		–	
	Animal-based meals (*n* 38) Ready meals (*n* 5) Home-cooked meals (*n* 33)	Up to supermarket shelf	530	440	474	232·2	0·17	< 0·0001*
		After cooking	541·1	430·9	491·8	229·5	0·22	
		GHGE % increase due to cooking	2·1 %		3·7 %		–	
	Plant-based meals (*n* 24) Ready meals (*n* 7) Home-cooked meals (*n* 17)	Up to supermarket shelf	150	225	253	185·2	0·83	
		After cooking	159·3	227·2	265·7	199·3	0·92	
		GHGE % increase due to cooking	6·1 %		4·0 %		–	
Electric stove cooked (Total gCO2e/100 g)	All meals (*n* 62) Ready meals (*n* 12) Homemade meals (*n* 50)	Up to supermarket shelf	435	472·5	395·5	252·2	0·72	–
		After cooking	451	474·9	409·7	238·5	0·89	
		GHGE % increase due to cooking	3·7 %		3·5 %		–	
	Animal-based meals (*n* 38) Ready meals (*n* 5) Homemade meals (*n* 33)	Up to supermarket shelf	530	440	474	232·2	0·17	< 0·0001*
		After cooking	543·8	428·7	495·8	226·3	0·22	
		GHGE % increase due to cooking	2·6 %		4·6 %		–	
	Plant-based meals (*n* 24) Ready meals (*n* 7) Home-cooked meals (*n* 17)	Up to supermarket shelf	150	225	253	185·2	0·83	
		After cooking	161·5	227·79	265·75	199·3	0·97	
		GHGE % increase due to cooking	7·6 %		5·1 %		–	
Microwave cooked (Total gCO2e/100 g)	All meals (*n* 39) Ready meals (*n* 39)	Up to supermarket shelf	480	700	–		–	–
		After cooking	483·1	700·2	–		–	
		GHGE % increase due to cooking	0·6 %		–		–	
	Animal-based meals (*n* 25) Ready meals (*n* 25)	Up to supermarket shelf	810	580	–		–	0·002*
		After cooking	817·8	588·3	–		–	
		GHGE % increase due to cooking	0·9 %		–		–	
	Plant-based meals (*n* 14) Ready meals (*n* 14)	Up to supermarket shelf	240	272·5	–		–	
		After cooking	246·1	271·5	–		–	
		GHGE % increase due to cooking	2·5 %		–		–	
Oven cooked (Total CO2e/100 g)	All meals (*n* 77) Ready meals (*n* 46) Home-cooked meals (*n* 31)	Up to supermarket shelf	510·5	640	479	234·5	0·04	–
		After cooking	607·3	686·5	518·9	280·1	0·0002*	
		GHGE % increase due to cooking	18·9 %		8·3 %		–	
	Animal-based meals (*n* 52) Ready Meals (*n* 32) Home-cooked meals (*n* 20)	Up to supermarket shelf	740	667·5	498	141·5	0·01	< 0·0001*
		After cooking	917·6	670·3	534·7	134·7	0·0004*	
		GHGE % increase due to cooking	24·0 %		7·3 %		–	
	Plant-based meals (*n* 25) Ready meals (*n* 14) Homemade meals (*n* 11)	Up to supermarket shelf	265	271	282	165·2	0·95	
		After cooking	401·4	254·1	300·7	176·8	0·07	
		GHGE % increase due to cooking	51·4 %		6·6 %		–	

GHGE, greenhouse gas emissions; gCO2e, gCO2 equivalents; N/A, not applicable; Prm-hc, *P*-value of difference between ready meals and equivalent home-cooked meals; Pab-pb, *P*-value of difference between animal-based and plant-based meals after cooking.

Data represent medians and interquartile ranges (IQR). None of the retrieved recipes for the homemade version of the meals reported the use of microwave. Cooking generally resulted in a significant increase in GHGE across all meals and cooking methods (*P* < 0·05).

* Statistical significance, adjusted using Bonferroni correction, was estimated at a *P*-value < 0·0027. No homemade meal recipe required the use of microwave, and hence no comparison among ready meals and homemade meals was feasible.

Overall, the most environmentally friendly and affordable (i.e. cheaper) products were plant-based home-prepared meals cooked on the gas or electric stove. Some examples include vegetable chow mein, ratatouille, spinach and potato curry with tomatoes and onion, vegetable curry or carrot and onion soup. In contrast, animal-based ready meals, either cooked in the oven or the microwave, produce the highest levels of GHGE and were the most expensive (online Supplementary Fig. 1).

## Discussion

This study explored how ready meals compared to equivalent home-cooked meals in terms of nutritional quality, GHGE and cost in dishes relevant to the UK market. All ready meals, but especially animal-based ready meals, had significantly higher levels of free sugars compared with equivalent home-cooked meals. In addition, ready meals had significantly higher GHGE than home-cooked meals up to the supermarket shelf, with cooking adding further GHGE, depending on the cooking method. Generally, ready meals costed significantly more (£0·30/100 g more) than their equivalent home-cooked meals. Animal-based oven-cooked ready meals had the highest levels of GHGE and were most expensive, whereas plant-based home-prepared meals cooked on the gas or electric stove had the lowest GHGE and costed least.

Diet-based studies have already shown that reductions in animal-based foods reduce GHGE, increase the nutritional quality and reduce the costs of total diets^(30)^ but thus far food-based studies are mostly lacking. No previous papers have compared ready meals to equivalent home-cooked meals in terms of nutritional quality, GHGE and/or cost; when such indicators were used, the research focused either on ready meals or home-cooked meals^(31,32)^. From a nutritional perspective, we did not find that ready meals were higher in salt, in line with one study^(33)^ whilst other studies did observe higher salt levels in ready meals^(18,34,35)^. However, it should be noted that the amount of salt added during cooking can vary, and furthermore, the salt content for home-cooked meals may be underestimated as many people cook with ‘salt to taste’ and may add more to the meal they consume. We did not find significant differences in the content of *trans*-fat, fibre and energy between ready meals and equivalent home-cooked meals, as has been observed in other studies^(18,19,33)^. This is relevant because previous studies have argued that increased consumption of ready meals was associated with a higher energy intake, poor compliance with national nutritional recommendations and abdominal obesity^(36)^.

Our data on GHGE is in line with those presented by Reynolds (2020)^(37)^, who found cooking to contribute between 8 and 84 % to total GHGE, with the environmental impact of cooking meats being higher than cooking vegetables. In our data, the main differences in GHGE align with the well-documented differences between plant- and animal-based meals, and differences due to the cooking method and cooking time, which are usually shorter for plant-based meals than animal-based meals. However, our data also showed that different cooking methods differentially contributed to GHGE, with oven cooking producing most GHGE, but with other cooking methods like gas and electric stove, and microwave cooking, contributing less than 10 % of total GHGE. In addition, recipes for the same type of meal can vary considerably, thereby affecting the environmental impact^(6)^. For example, replacing meat with soya and seitan could reduce the environmental impact by up to 27 %^(6)^. In our study, homemade meals were cheaper, had lower GHGE and had a better nutritional quality up to a supermarket shelf, which may be partly due to differences in nutrient composition.

Large food producers and supermarkets can influence the way we eat by offering healthier, more environmentally sustainable and affordable choices. The sector of plant-based meals is currently the fastest-growing food category in the UK, with a growth of 92 % since 2018^(38)^. Furthermore, a recent study highlighted that adequate labelling of ready meals could help improve food consumption-related climate change and health issues^(39)^. Therefore, cooking instructions on the back of the packaging may encourage consumers to choose the cooking method causing the least GHGE. Using cooking methods such as slow cookers, pressure cookers and microwaves, all of which have a lower energy use, would significantly lower GHGE from home-cooking^(27)^.

The strengths of this study include considering three dimensions (nutritional quality indicators, GHGE and cost) that are important for healthy, environmentally sustainable and affordable food choices. Previous studies have considered the estimation of GHGE of recipes or meals^(37,40,41)^; however, our current analysis also includes the cost of the meals. We also studied a larger number of ready meals and equivalent home-cooked meals than many previous studies, particularly concerning their GHGE^(33)^. We also considered the differences in GHGE and cost across animal- and plant-based meals, which is relevant considering the importance of moving towards less meat-intensive diets in order to reduce GHGE^(39)^. Lastly, this study used more up-to-date data than previous studies, which is essential as ready meals are constantly being reformulated based on salt and sugar reduction targets.

An important limitation of this study is that we did not include the cost of reheating or cooking in our analysis. This is important considering that home-cooked meals can be up to six times more expensive after cooking than ready meals^(18,33)^, and consumers may purchase ready meals because these are quicker and cheaper to (re)heat in the microwave. Thus, whilst we found that ready meals, overall and those that are plant-based, cost significantly more than equivalent home-cooked meals, based on a large selection of meals, a previous study found that ready meals were no more expensive than buying the ingredients for home-cooked meals when considering the ten most frequently purchased ready meals in a sample of Scottish households^(33)^. Furthermore, we did not consider the cost of household labour, which might be typically valued at the wage level of the household meal preparer, and this could be significant^(42)^. These issues will need to be interpreted in the context of additional cooking costs. Another limitation of our study is that we did not examine artificial preservatives, stabilisers, colourings or flavours as part of the nutritional quality of the meals^(43)^. Also, no side dishes were considered, and all our measurements were expressed per 100 g of the meal. Thus, we did not consider the possible differences in portion sizes between home-cooked meals and ready meals. For example, ready meals are typically bought to provide for one or sometimes two portions, whilst home-cooked meals are often prepared as multiple portions. As in our study, values of GHGE were expressed per 100 g of product, and our calculations may have led to relatively higher GHGE (per 100 g of product) for ready meals, compared with equivalent home-cooked meals.

In conclusion, whilst the purchase and consumption of ready meals in the UK has increased in the past years, homemade meals have better nutritional characteristics, are cheaper and have lower GHGE, especially those that are plant-based. However, cooking can add to GHGE and the cost of preparing a ready or home-cooked meal, and better dissemination of this information to the consumer could potentially lead to more healthy, sustainable and affordable meal choices.

## Supplementary material

For supplementary material accompanying this paper visit https://doi.org/10.1017/S1368980023000034. click here to view supplementary material

## References

[1] Monteiro CA , Cannon G , Moubarac JC et al. (2018) The UN Decade of Nutrition, the NOVA food classification and the trouble with ultra-processing. Public Health Nutr 21, 5–17. 28322183 10.1017/S1368980017000234 PMC10261019

[2] Chen X , Zhang Z , Yang H et al. (2020) Consumption of ultra-processed foods and health outcomes: a systematic review of epidemiological studies. Nutr J 19, 86. 32819372 10.1186/s12937-020-00604-1 PMC7441617

[3] Seferidi P , Scrinis G , Huybrechts I et al. (2020) The neglected environmental impacts of ultra-processed foods. Lancet Planet Health 4, e437–e438. 33038314 10.1016/S2542-5196(20)30177-7

[4] O’Mahony A (2020) The Break-Up: Ready Meals Category Report 2020. London: The Grocer.

[5] Mintel (2019) Ready Meals and Ready-to-Cook Foods – UK – July 2019. store.mintel.com (accessed October 2021).

[6] Rivera XCS & Azapagic A (2019) Life cycle environmental impacts of ready-made meals considering different cuisines and recipes. Sci Total Environ 660, 1168–1181. 30743912 10.1016/j.scitotenv.2019.01.069

[7] De Boer M , McCarthy M , Cowan C et al. (2004) The influence of lifestyle characteristics and beliefs about convenience food on the demand for convenience foods in the Irish market. Food Qual Preference 15, 155–165.

[8] Jabs J & Devine CM (2006) Time scarcity and food choices: an overview. Appetite 47, 196–204. 16698116 10.1016/j.appet.2006.02.014

[9] Rivera XCS , Orias NE & Azapagic A (2014) Life cycle environmental impacts of convenience food: comparison of ready and home-made meals. J Cleaner Prod 73, 294–309.

[10] Sonesson U , Mattsson B , Nybrant T et al. (2005) Industrial processing *v.* home cooking: an environmental comparison between three ways to prepare a meal. AMBIO: A J Hum Environ 34, 414–421. 10.1639/0044-7447(2005)034[0414:ipvhca]2.0.co;2 16092278

[11] Pechey R & Monsivais P (2016) Socioeconomic inequalities in the healthiness of food choices: exploring the contributions of food expenditures. Prev Med 88, 203–209. 27095324 10.1016/j.ypmed.2016.04.012 PMC4910945

[12] Courtney Scott JS & Taylor A (2018) Affordability of the UK’s Eatwell Guide. The Food Foundation. https://foodfoundation.org.uk/sites/default/files/2021-10/Affordability-of-the-Eatwell-Guide_Final_Web-Version.pdf (accessed October 2022).

[13] Jones NRV , Tong TYN & Monsivais P (2018) Meeting UK dietary recommendations is associated with higher estimated consumer food costs: an analysis using the National Diet and Nutrition Survey and consumer expenditure data, 2008–2012. Public Health Nutr 21, 948–956. 29198220 10.1017/S1368980017003275 PMC5848749

[14] Team FS (2016) Food Statistics Pocketbook 2016. York: Department for Environment, Food and Rural Affairs.

[15] England PH (2021) National Diet and Nutrition Survey. London: Public Health England.

[16] McCance RA & Widdowson EM (2021) McCance and Widdowson’s The Composition of Foods Integrated Dataset 2021: User Guide. London: Public Health England.

[17] Research NS (2021) National Diet and Nutrition Survey Years 1–11, 2008–2019, 19th ed. https://assets.publishing.service.gov.uk/government/uploads/system/uploads/attachment_data/file/943114/NDNS_UK_Y9-11_report.pdf (accessed October 2022).

[18] Howard S , Adams J & White M (2012) Nutritional content of supermarket ready meals and recipes by television chefs in the United Kingdom: cross sectional study. BMJ: Br Med J 345, e7607. 23247976 10.1136/bmj.e7607 PMC3524368

[19] Remnant J & Adams J (2015) The nutritional content and cost of supermarket ready-meals. Cross-sectional analysis. Appetite 92, 36–42. 25963106 10.1016/j.appet.2015.04.069 PMC4509783

[20] TESCO (2012) Product Carbon Footprint Summary. UK: TESCO.

[21] Bates RL , Chambers NG & Craig LCA (2019) Greenhouse gas emissions of UK diets. Proc Nutr Soc 78, E65.

[22] Aceves-Martins M , Bates RL , Craig LCA et al. (2022) Nutritional quality, environmental impact and cost of ultra-processed foods: a UK food-based analysis. Int J Environ Res Public Health 19, 3191. 35328877 10.3390/ijerph19063191 PMC8948822

[23] Department for Environment FRA (2013) 2012 Greenhouse Gas Conversion Factors for Company Reporting. London: British Government.

[24] BSI (2011) Specification for the Assessment of the Life Cycle Greenhouse Gas Emissions of Goods and Services. London: BSI.

[25] BBC (2022) BBC Good Food. https://www.bbcgoodfood.com/ (accessed September 2021).

[26] Sainsbury’s (2022) Sainsbury’s Recepies. https://recipes.sainsburys.co.uk/ (accessed September 2021).

[27] Frankowska A , Rivera XS , Bridle S et al. (2020) Impacts of home cooking methods and appliances on the GHG emissions of food. Nat Food 1, 787–791. 10.1038/s43016-020-00200-w 37128063

[28] Department for Business EaIS (2021) 2019 UK Greenhouse Gas Emissions National Statitistics. London: Department for Business, Energy and Industrial Strategy.

[29] González-García S , Esteve-Llorens X , Moreira MT et al. (2018) Carbon footprint and nutritional quality of different human dietary choices. Sci Total Environ 644, 77–94. 29981520 10.1016/j.scitotenv.2018.06.339

[30] Willits-Smith A , Aranda R , Heller MC et al. (2020) Addressing the carbon footprint, healthfulness, and costs of self-selected diets in the USA: a population-based cross-sectional study. Lancet Planet Health 4, e98–e106. 32220679 10.1016/S2542-5196(20)30055-3 PMC7232940

[31] Mills S , Brown H , Wrieden W et al. (2017) Frequency of eating home cooked meals and potential benefits for diet and health: cross-sectional analysis of a population-based cohort study. Int J Behav Nutr Phys Act 14, 1–11. 28818089 10.1186/s12966-017-0567-y PMC5561571

[32] Wolfson JA , Willits-Smith AM , Leung CW et al. (2022) Cooking at home, fast food, meat consumption, and dietary carbon footprint among US adults. Int J Environ Res Public Health 19, 853. 35055675 10.3390/ijerph19020853 PMC8775624

[33] Naruseviciute G , Whybrow S , Macdiarmid J et al. (2015) Is ‘home cooked’ healthier and cheaper than ready meals? Proc Nutr Soc 74, E90.

[34] Kanzler S , Manschein M , Lammer G et al. (2015) The nutrient composition of European ready meals: protein, fat, total carbohydrates and energy. Food Chem 172, 190–196. 25442542 10.1016/j.foodchem.2014.09.075

[35] Kanzler S , Hartmann C , Gruber A et al. (2014) Salt as a public health challenge in continental European convenience and ready meals. Public Health Nutr 17, 2459–2466. 24809795 10.1017/S1368980014000731 PMC10282222

[36] Alkerwi AA , Crichton GE & Hébert JR (2015) Consumption of ready-made meals and increased risk of obesity: findings from the Observation of Cardiovascular Risk Factors in Luxembourg (ORISCAV-LUX) study. Br J Nutr 113, 270–277. 25488071 10.1017/S0007114514003468 PMC4302389

[37] Reynolds C (2020) Sustainable Gastronomy: The Environmental Impacts of How We Cook Now and How the “Sustainable Diets” Agenda Might Shape How We Cook in the Future? https://openaccess.city.ac.uk/id/eprint/24232/ (accessed October 2022).

[38] Better E (2021) Ready Meals 2021 Snapshot Survey. Eating Better. https://www.eating-better.org/uploads/Documents/2021/EB-ready-meals-survey-FINALJune2021.pdf (accessed October 2022).

[39] Macdiarmid JI , Cerroni S , Kalentakis D et al. (2021) How important is healthiness, carbon footprint and meat content when purchasing a ready meal? Evidence from a non-hypothetical discrete choice experiment. J Cleaner Prod 282, 124510.

[40] Reynolds C , Takacs B , Klimashevskaia A et al. (2021) Comparing the Environmental Impacts of Recipes from Four Different Recipe Databases Using Natural Language Processing. https://openaccess.city.ac.uk/id/eprint/27235/ (accessed October 2022).

[41] Reynolds C (2022) The Evolution of ‘Sustainable’ and Vegetarian Recipes from Manuscripts and Cookbooks to Online: Their Environmental Impact, and What this Means for the Future. https://openaccess.city.ac.uk/id/eprint/27676/ (accessed October 2022).

[42] Rose D (2007) Food stamps, the Thrifty Food Plan, and meal preparation: the importance of the time dimension for US nutrition policy. J Nutr Educ Behavior 39, 226–232. 10.1016/j.jneb.2007.04.180 17606249

[43] Johnson EM , Jung DY-G , Jin DY-Y et al. (2018) Bacteriocins as food preservatives: challenges and emerging horizons. Crit Rev Food Sci Nutr 58, 2743–2767. 28880573 10.1080/10408398.2017.1340870

