# Supplementary material for: Ready meals, especially those that are animal-based and cooked in an oven, have lower nutritional quality and higher greenhouse gas emissions and are more expensive than equivalent home-cooked meals
Source: Public Health Nutr. 2023 Jan 17;26(3):531–9. doi: 10.1017/S1368980023000034 (PMC9989702; doi:10.1017/S1368980023000034)
Supplement: Supplementary file 1 [file S1368980023000034sup001.docx]

**Supplementary Table 1.** Results of Shapiro–Wilk tests

| **Indicator** | | **Shapiro–Wilk tests result** | **P-value** |
| --- | --- | --- | --- |
| Total Carbohydrates (g/100g) | | W = 0.93493 | 5.037e-05 |
|  | Free sugars (g/100g) | W = 0.59163 | 7.286e-16 |
| Total Protein (g/100g) | | W = 0.97216 | 0.02288 |
| Total Fat (g/100g) | | W = 0.86866 | 2.469e-08 |
|  | Trans Fat (g/100g) | W = 0.9024 | 8.257e-07 |
| Fibre (g/100g) | | W = 0.97976 | 0.099 |
| Salt (g/100g) | | W = 0.94788 | 0.0003439 |
| Kilocalories (kcal/100g) | | W = 0.8999 | 6.227e-07 |
| GHGE up to supermarket shelf (gCO2e/100 g) | | W = 0.89487 | 3.568e-07 |
|  | Gas Stove cooked – (gCO2e /100 g) | W = 0.89993 | 8.995e-05 |
|  | Electric Stove cooked – (gCO2e /100 g) | W = 0.89912 | 8.389e-05 |
|  | Microwave cooked – (gCO2e /100 g) | W = 0.89088 | 0.001221 |
|  | Oven cooked – (gCO2e /100 g) | W = 0.89156 | 7.79e-06 |
| Total cost (GBP /100 g) | | W = 0.93446 | 4.711e-05 |

Significance estimated at a p-value < 0.05


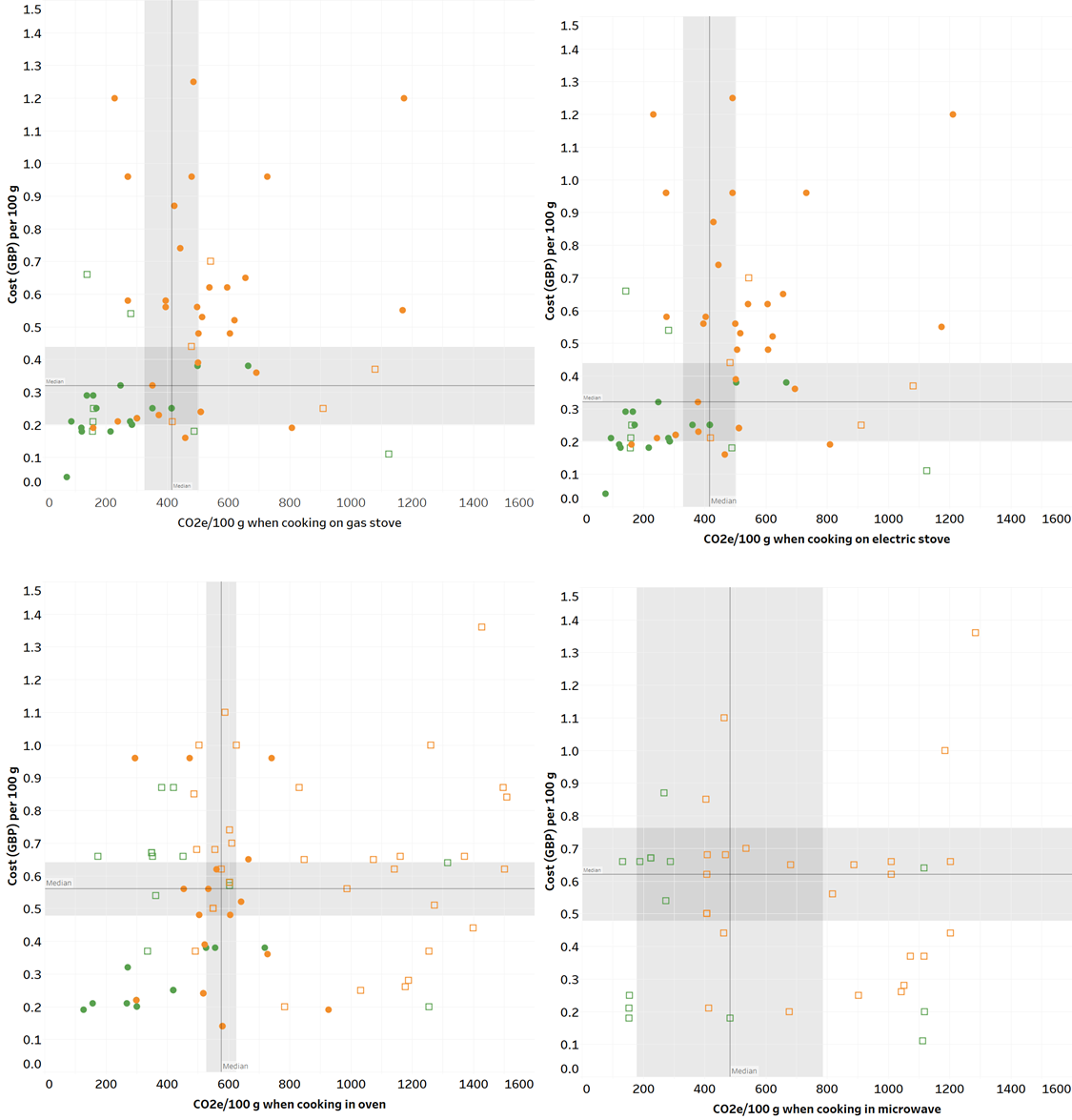


**Supplementary Figure 1**. Comparison of GHGE and cost of ready meals and equivalent home-cooked meals, either animal or plant-based. ready meal ⚫ home-cooked meal, ◼⚫ animal-based meals, ◼⚫ plant-based meals. CO2e - gCO2-equivalents. GBP-Great British pound £. Grey lines show the median and 95% Confidence Interval. Gas and electric stove cooked meals: n=62 (n=12 ready meals of which 5 animal-based and 7 plant-based; and n=50 home-cooked meals of which 33 animal-based and 17 plant-based). Microwave cooked meals: n=39 ready meals, of which 25 were animal-based and 14 plant-based. Oven cooked meals: n=77 (n=46 ready meals of which 52 animal-based and 25 plant-based; and n=31 home-cooked meals of which 20 animal-based and 11 plant-based).
